# Pegylated Trastuzumab Fragments Acquire an Increased *in Vivo* Stability but Show a Largely Reduced Affinity for the Target Antigen

**DOI:** 10.3390/ijms17040491

**Published:** 2016-04-01

**Authors:** Fabio Selis, Giuseppina Focà, Annamaria Sandomenico, Carla Marra, Concetta Di Mauro, Gloria Saccani Jotti, Silvia Scaramuzza, Annalisa Politano, Riccardo Sanna, Menotti Ruvo, Giancarlo Tonon

**Affiliations:** 1 Bioker srl-Multimedica Group, c/o Institute of Genetics and Biophysics, National Research Council (CNR-IGB) via P. Castellino 111, 80131 Napoli, Italy; carlamarra78@yahoo.it (C.M.); silvia.scaramuzza@multimedica.it (S.S.); annalisa.politano@gmail.com (A.P.); riccardo.sanna@multimedica.it (R.S.); giancarlo.tonon@multimedica.it (G.T.); 2 Institute of Biostructure and Bioimaging, National Research Council (IBB-CNR), via Mezzocannone 16, 80134 Napoli, Italy; giuseppina.foca@gmail.com (G.F.); menotti.ruvo@unina.it (M.R.); 3 Centro Interuniversitario di Ricerca sui Peptidi Bioattivi (CIRPeB), University of Naples Federico II, via Mezzocannone 16, 80134 Napoli, Italy; 4 Department of Clinical Medicine and Surgery, University of Naples Federico II, 80134 Napoli, Italy; dimauro.co@alice.it; 5 Department of Biomedical, Biotechnological and Translational Science (S.Bi.Bi.T.), Università di Parma, 43126 Parma, Italy; gloria.saccani@unipr.it

**Keywords:** PEGylation, antibody fragment, Fab, papain digestion, pepsin digestion, pharmacokinetics

## Abstract

PEGylation of biomolecules is a major approach to increase blood stream half-life, stability and solubility of biotherapeutics and to reduce their immunogenicity, aggregation potential and unspecific interactions with other proteins and tissues. Antibodies have generally long half-lives due to high molecular mass and stability toward proteases, however their size lowers to some extent their potential because of a reduced ability to penetrate tissues, especially those of tumor origin. Fab or otherwise engineered smaller fragments are an alternative but are less stable and are much less well retained in circulation. We have here investigated the effects of various PEGylations on the binding properties and *in vivo* half-life of Fab fragments derived from the enzymatic splitting of Trastuzumab. We find that PEGylation increases the half-life of the molecules but also strongly affects the ability to recognize the target antigen in a way that is dependent on the extent and position of the chemical modification. Data thus support the concept that polyethylene glycol (PEG) conjugation on Trastuzumab Fabs increases half-life but reduces their affinity and this is a fine balance, which must be carefully considered for the design of strategies based on the use of antibody fragments.

## 1. Introduction

Human epidermal growth factor receptor 2 (HER2) positive breast cancer accounts for 20%–30% of all breast cancers and has the second-poorest prognosis among breast cancer subtypes. Trastuzumab (Herceptin^®^) is a humanized IgG1-*κ* light chain mAb in which the complementary-determining regions (CDR) of a HER2-specific mouse mAb were joined to human antibody framework regions through genetic engineering [1,2]. The approval of Trastuzumab in 1998 significantly improved patients’ outcomes and paved the way to targeted approaches in breast cancer treatment [3,4,5]. Since then a number of other mAbs have been clinically approved for cancer therapy, together with a small number of antibody fragments, mostly Fabs [6,7]; several other classes of engineered antibody fragments are also in development [6,8]. It is well known that the Fc portion of a human IgG is important for keeping antibody stability and thus ensure high serum levels following intravenous administration. Indeed, intact IgGs are more stable in serum and have a longer half-life compared to the corresponding functional fragments [9,10]. On the other hand, smaller fragments have the advantage of penetrating more deeply into tissues, especially those of cancerous nature [11], thus diffusing more efficiently toward the molecular antigens they are targeted to. Furthermore, Fc-free antibody fragments have no complications deriving from Fc receptor engagement and activation and no side effects deriving from Antibody-dependent cell-mediated cytotoxicity (ADCC). In addition, capture by the high affinity Fc receptors does subtract the active antibodies from circulation thus reducing their concentration and their potential. A balance between such two opposite requirements, reducing the rapid clearance and removal of unneeded protein portions, is not restricted to therapeutic antibodies, but is ever more frequently necessary for other types of biotherapeutics. One elegant option is represented by the covalent attachment of polyethylene glycol (PEG). PEG mostly masks protein surface protecting them from the immune system and proteases. At the same time it increases protein volume preventing or retarding excretion from kidneys. Therefore, biotherapeutics PEGylation has several advantages over other chemical modifications because of the very poor antigenicity of PEG and the availability of a number of reagents ad hoc developed for site specific protein modification under very mild conditions [12]. PEGylation also protects proteins from proteolytic degradation, contributing to enhance their half-life and, most importantly, reduces non-specific interactions hardly suppressing aggregation and increasing solubility. PEG has been thereby largely used to develop various commercially available protein derivatives to prolong their half-life and to reduce immunogenicity while maintaining their activity [13,14,15].

In this work we have evaluated the effects of PEGylation on the activity and half-life of antibody fragments obtained by proteolytic hydrolysis. As model antibody we have used Trastuzumab, which is one of the first antibody that has reached the market and is therefore one of the best characterized from a functional and structural point of view. For this investigation we have envisaged two alternative strategies of PEG-derivatisation. In one case we generated C-terminally-derivatized Fabs bearing 10 and 20 kDa PEG on pepsin generated free cysteines on the antibody heavy chain. In the other case, we studied the effects produced by a more invasive N-terminal modification which, being very close to the antibody CDR, can more extensively prevent antigen recognition. The complete list of reagents generated and investigated is reported under the section of Methods.

To determine how PEGylation on the different sites can affect the antibody recognition of its antigen, the HER2 receptor, binding studies have been conducted by surface plasmon resonance (SPR) using a Biacore 3000 instrument, determining kinetic and thermodynamic parameters underlying the interactions. Comparative binding studies have also been performed by ELISA. Preliminary pharmacokinetic profiles of some selected PEG derivatives and the intact antibody have been obtained following single intravenous (*i.v.*) administration in mice. Results mostly show that PEGylation can be used to increase the half-lives of antibody fragments but the modification strongly affects antigen recognition. To the best of our knowledge, this is the first study performed on a broad set of PEG-Trastuzumab derivatives providing both binding and pharmacokinetic comparative data.

## 2. Results

**Figure 1:**
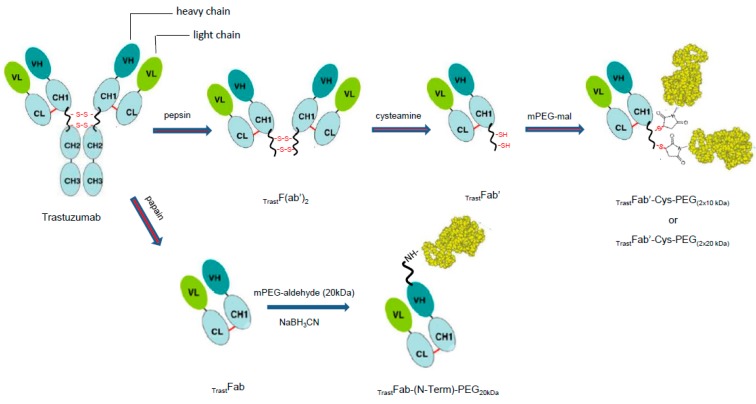
Schematic representation of all the antibody fragments and PEGylated variants prepared starting from Trastuzumab, reported as _Trast_F(ab’)_2_, _Trast_Fab’, _Trast_Fab, _Trast_Fab’–Cys–PEG_(2 × 10 kDa)_, _Trast_Fab’–Cys–PEG_(2 × 20 kDa)_ and _Trast_Fab–(N-Term)–PEG_20 kDa_. VH and VL are variable domains of the antibody heavy and light chain respectively; CL is the constant domain of the antibody light chain; CH1, CH2 and CH3 are the constant domains of the antibody heavy chain.

A list of all antibody reagents used in this study is reported in Table 1 together with the expected molecular weight (*M*w). In Figure 1, cartoons of the generated fragments are reported. The experimental conditions for their generation are reported in the section materials and methods, after the Discussion.

### 2.1. Preparation of _Trast_Fab

**Figure 2:**
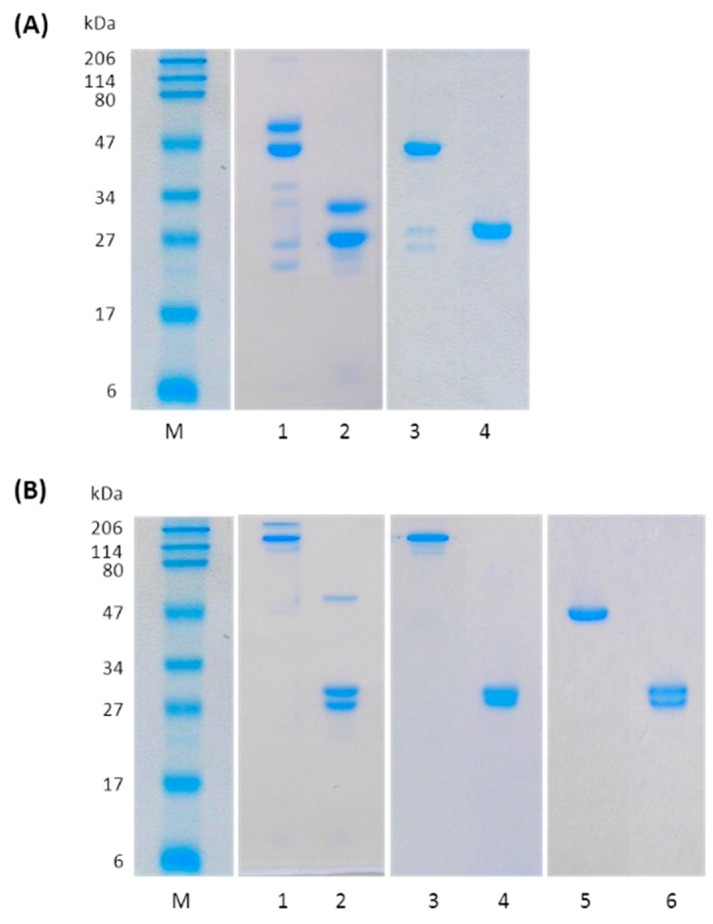
SDS-PAGE analysis (15% separating gel) of Fab fragments obtained by proteolytic digestion of Trastuzumab with papain or pepsin: (**A**) Proteolysis of Trastuzumab with papain: protein standards (line M); reaction mixture after 90 min incubation at 37 °C under non reducing (lane 1) and reducing conditions (lane 2); and purified _Trast_Fab under non reducing (lane 3) and reducing conditions (lane 4); (**B**) Proteolysis of trastuzumab with pepsin: protein standards (line M); reaction mixture after 16 h incubation at 37 °C under non reducing (lane 1) and reducing conditions (lane 2); purified _Trast_F(ab’)_2_ under non reducing (lane 3) and reducing conditions (lane 4); and purified _Trast_Fab’ under non reducing (lane 5) and reducing conditions (lane 6). Proteins were visualized by Bio-Safe Coomassie blue stain. Reduction was achieved by treating samples with 2-mercaptoethanol before loading.2.2. N-terminal _Trast_Fab PEGylation

_Trast_Fab was produced by a standard proteolytic cleavage with papain (Figure 2A, lanes 1, 2), splitting the antibody into two Fab units and an Fc portion [17]. After 90 min incubation, the mixture was firstly purified by affinity chromatography eluting as expected the Fab fragment in the unbound fraction.

The purified Fab showed a molecular weight of about 48 kDa by SDS-PAGE analysis under non-reducing conditions, while under reducing conditions, the separated light and heavy chains were detected as homogenous bands at ~25 kDa (Figure 2A, lanes 3, 4). After purification, an overall 25% yield was achieved in typical preparations.

**Figure 3:**
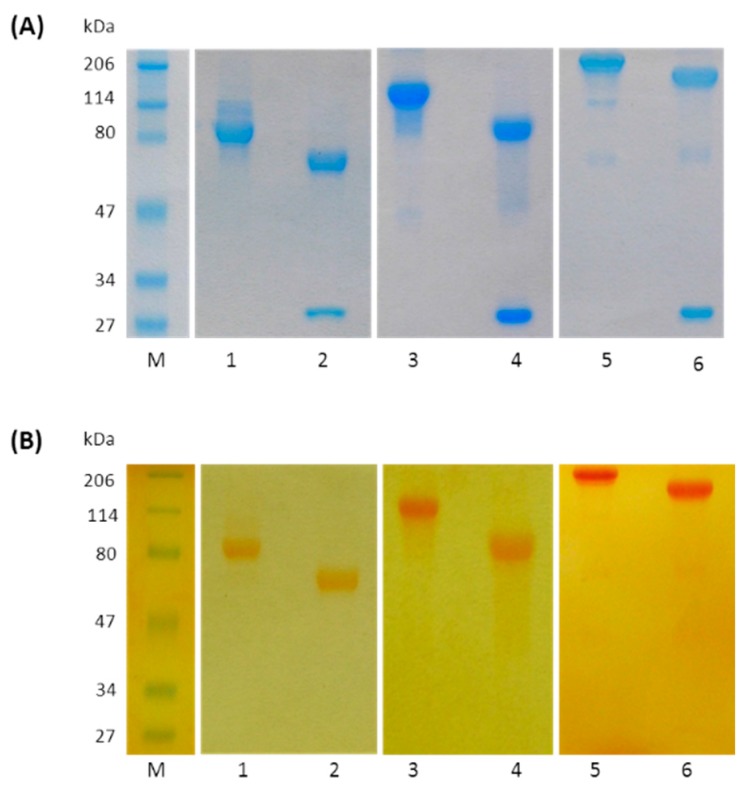
SDS-PAGE analysis (10% separating gel) of PEGylated Fab fragments. PEGylated Fab fragments were detected with Bio-Safe Coomassie stain (**A**) and with iodine stain (**B**) protein standards (lane M); purified _Trast_Fab-(N-Term)-PEG_20 kDa_ under non reducing (lane 1) and reducing conditions (lane 2); purified _Trast_Fab’-Cys-PEG_(2 × 10 kDa)_ under non reducing (lane 3) and reducing conditions (lane 4); and purified _Trast_Fab’-Cys-PEG_(2 × 20 kDa)_ under non reducing (lane 5) and reducing conditions (lane 6). Reduction was achieved by treating samples with 2-mercaptoethanol before loading.

Fab generated by papain digestion of trastuzumab was reacted with 20 kDa PEG-propionaldehyde (PEG-aldehyde) to form the mono conjugated Fab at the N-terminus. The reaction was conducted using slightly acidic condition (acetate buffer pH 4.5) to favor PEGylation to the N-terminal amino groups. The two-fold molar excess of PEG-aldehyde yielded predominantly the mono-PEGylated Fab as determined by SE chromatography analysis (Figure S1). Multiple PEGylated forms were present in only low amount. After purification by cation exchange chromatography, _Trast_Fab-(N-Term)-PEG_20 kDa_ showed an apparent molecular mass of 85 kDa upon reduction conditions instead of about 70 kDa (50 kDa for the Fab and 20 kDa for the PEG). The higher *M*w was due to the PEG high hydrodynamic radius. Upon reduction, we stained a band of about 60–65 kDa for the PEG conjugated heavy chain and 27 kDa for the non-PEGylated light chain (see lanes 1, 2 with coomassie stain (Figure 3A) and with iodine stain (Figure 3B). The fully reduced light chain migrated as a slightly higher *M*w molecule likely due to its elongated shape. Regarding the PEGylated heavy chain, it has been reported, that PEG interacts with SDS micelles and that PEGylation decreases the mobility of the protein [18,19,20], therefore, the apparent molecular weight of a PEGylated protein on SDS gels appears as having a much higher *M*w. The apparent volume of macromolecules can be roughly derived from the method proposed by Fee and Alstine, and using as a first approximation the *a* and *b* constants of albumin [16]. By this approach an unconjugated Fab has an apparent volume of about 163 nm^3^, a Fab conjugated with a 20 kDa PEG has a volume of about 700 nm^3^, whereas a Fab conjugated with a 40 kDa PEG has a volume of about 2000 nm^3^. This strongly indicates that increasing the number of PEG moieties strongly impacts the relative molecular size and the ability to migrate in a SDS gel or in gel filtration matrix of these macromolecules.

The hydrodynamic effective size of each molecule was determined using a Zorbax GF-250 (4.6 mm × 250 mm) column equilibrated at 0.3 mL/min in 0.063 M phosphate buffer pH 7.3, containing 3% (*v/v*) Isopropanol. The column was kept at room temperature, and the absorbance was monitored at 215 nm. Molecular weight standards containing myoglobin, ovalbumin, γ-globulin, ferritin and thyroglobulin were used to generate a standard curve from which the effective size of the PEGylated antibodies was estimated.

The apparent MW dramatically increased relative to the theoretical MW with the attachment of large PEG moieties to the Fab molecule (Table 1).

Western blotting, performed with an anti-human Fab antibody specific for light chain, of the mono-PEGylated Fab under reducing conditions, showed that conjugation occurred only on the heavy chain (see Figure S2). Under the reaction conditions used (acetate buffer pH 4.5), the preferred nucleophile is the α-amino group (N-terminus) [21].2.3. Preparation of _Trast_Fab’ and _Trast_F(ab’)_2_

Trastuzumab was converted to _Trast_F(ab’)_2_ by proteolytic digestion using pepsin with typical yields of 87% as estimated by SE-HPLC (size exclusion high performance liquid chromatography) analyses (Figure S3) and SDS-PAGE (see lanes 1 and 2 in Figure 2B). _Trast_F(ab’)_2_ was purified to homogeneity by cation exchange chromatography using a saline gradient and showed an apparent mass by SDS-PAGE of 110 kDa under non-reducing conditions. Upon reduction, the product was stained as 27 kDa bands (see lanes 3 and 4 in Figure 2B).

_Trast_F(ab’)_2_ fragment was reduced to _Trast_Fab’ by cysteamine treatment. Reduction occurred after 16 h reaction at room temperature with a yield of 90% as estimated by SE-HPLC analyses (Figure S4). After reduction, _Trast_Fab’ was purified by gel filtration. Analysis by SDS-PAGE and SEC-HPLC of _Trast_Fab’ confirmed the integrity of the disulfide bridge between the heavy and the light chains of the Fab, indeed the Fab’ had an apparent mass by SDS-PAGE of 45 kDa under non-reducing conditions collapsing into 25 kDa bands upon reduction (see lanes 5 and 6 in Figure 2B).

### 2.4. Preparation of di-PEGylated _Trast_Fab’

Methoxypolyethylene glycol maleimide (MeO-PEG-maleimide) of molecular weight 10 and 20 kDa was attached to the reduced _Trast_Fab’. The di-PEGylated derivatives were obtained with a yield of about 70% calculated on the basis of initial amount of the Fab fragment, as estimated by SE-HPLC analyses (Figure S5). The two conjugates, _Trast_Fab’-Cys-PEG_(2 × 10 kDa)_ and _Trast_Fab’-Cys-PEG_(2 × 20 kDa)_, were purified to homogeneity by cation exchange chromatography. Due to the shielding of PEG, the di-PEGylated product eluted at shorter retention times followed by the mono PEGylated and the unconjugated Fab’, respectively [22]. The unconjugated PEG eluted during the step of loading because it is uncharged. Both purified di-PEGylated products were analyzed by SDS-PAGE, showing a molecular mass of 200 and 150 kDa for _Trast_Fab’-Cys-PEG_(2 × 20 kDa)_ and _Trast_Fab’-Cys-PEG_(2 × 10 kDa)_, respectively (see lanes 3 and 4 for _Trast_Fab’-Cys-PEG_(2 × 10 kDa)_ and lanes 5 and 6 for _Trast_Fab’-Cys-PEG_(2 × 20 kDa)_ with coomassie stain (Figure 3A) and with iodine stain (Figure 3B)). The size of the PEG-Fab was consistent with predictions based on the large hydrodynamic volume of PEG relative to mass (Table 1). The elongated structure of PEG typically leads to about a 10-fold difference in mass *versus* apparent size [23]. While this clearly can lessen tumor penetration, the reduced interaction of antibody and fragments with tissues and other biomolecules can largely improve this feature.

### 2.5. ELISA Binding Studies

The affinity of Fab fragments and PEGylated Fab fragments for ErbB2/HER2 was determined by ELISA. The assay was carried out coating the receptor and detecting the bound Fab derivatives using anti-Fab antibodies. As show in Figure S6, saturating curves were obtained for all binding experiments, though at different concentrations. In Table 2, the values of *EC*_50_, which reflect the relative affinity of the different molecules for the receptor, are reported.

We observed that the affinity of _Trast_Fab, _Trast_Fab’ and _Trast_F(ab’)_2_ for ErbB2/HER2 was similar to that of Trastuzumab, while comparing the affinity of PEGylated fragments with the corresponding unPEGylated variants, we obtained in all cases a significant reduction of binding affinity, most likely due to the steric interference of PEG with the antigen-binding site (see Table 2). Attachment of the 20 kDa MeO-PEG at the N-terminus resulted in a two-fold binding affinity loss compared with the unconjugated _Trast_Fab (about 0.8 nM compared to about 0.4 nM). Compared to _Trast_Fab’, _Trast_Fab’-Cys-PEG_(2 × 10 kDa)_ and _Trast_Fab’-Cys-PEG_(2 × 20 kDa)_ underwent a three-fold and two-fold affinity loss, respectively.

### 2.6. Surface Plasmon Resonance (SPR) Binding Measurements

SPR analyses were carried out to compare the binding properties of the Trastuzumab-derived new products with those of the native antibody. *K*_D_ values, (the affinity constant value, which is equal to the equilibrium dissociation constant (*K*_D_) derived from the ratio between the rate dissociation constant (*k*_off_ or *K*_d_) and association constant (*k*_on_ or *K*_a_), were determined for each analyte on three distinct channels at three different immobilization levels. Final values were determined as the average of the *K_D_*s extrapolated from each channel at every concentration; values exhibiting a *t*-value less than 10 were excluded from the average. Such values typically denote a mass-transfer effect, commonly detected in the presence of large-size molecules, including antibodies.

The interaction of the full-size antibody to ErbB2/HER2 receptor was initially investigated by injecting Trastuzumab in a range of increasing concentrations between 0.06 and 3 nM. The interaction of the whole antibody with the antigen was characterized by an overall *K*_D_ value of 0.13 nM. The kinetic and thermodynamic binding constants underlying the interactions between the antibody-derived fragments and the immobilized receptor were similarly determined and are summarized in Table 3. Overlaid sensorgrams and the related kinetic and thermodynamic parameters determined for each injection are reported in Figures S7–S13. As can be seen, _Trast_Fab and _Trast_Fab’ displayed a *K*_D_ value of 0.40 and 0.29 nM, respectively, very similar to that of the whole antibody, thus suggesting that the binding affinity was essentially retained when the immunoglobulin was reduced into smaller functional fragments [15].

Remarkably, conjugation of 20 kDa PEG at the N-terminus of the Fab (_Trast_Fab-(N-Term)-PEG_20 kDa_), produced an expected strong decrease of binding affinity (4.82 nM), estimated as almost 13-fold compared to the unPEGylated _Trast_Fab. This was likely due to a shielding effect of the binding site by the polymer, which is on a crucial region for antigen recognition. This effect was confirmed by the slower association rate (*k*_a_) and the faster dissociation rate (*k*_d_) which are indicative of a poorer accessibility of the binding analyte.

In contrast, _Trast_Fab’-Cys-PEG_(2 × 10 kDa)_, corresponding to the _Trast_Fab’ derivative PEGylated at the C-terminus with two 10 kDa PEG tails, exhibited a lower *K*_D_ (0.82 nM) compared with the N-terminally PEGylated product. This value was however 3-fold higher than the one determined for the non PEGylated fragment. Such affinity reduction was essentially ascribed to a slower association rate compared to the unconjugated fragment (the dissociation rate was instead comparable, see Table 3), suggesting that the PEG loading slowed down the molecule delivery toward the immobilized receptor, whereas it did not affect the dissociation [24]. The Fab conjugated with two 20 kDa PEG chains was found to possess a *K*_D_ of 2.24 nM, an affinity decrease of about 12-fold compared to the unconjugated _Trast_Fab’, a result in line with the concept that although the PEG did not interfere with the antigen binding site, its presence influenced the rate of approaching the immobilized receptor by the conjugated antibody fragment.

The _Trast_F(ab’)_2_ fragment, retaining both Fab arms, displayed a behavior similar to that of the whole antibody, indeed it displayed a *K*_D_ value comparable to that of Trastuzumab (0.18 nM compared to 0.13 nM), resulting from a combination of both similar association and dissociation rates, and also provided comparable signal in terms of RU (Response Units) at about the same concentrations.

### 2.7. Pharmacokinetic Properties

**Figure 4:**
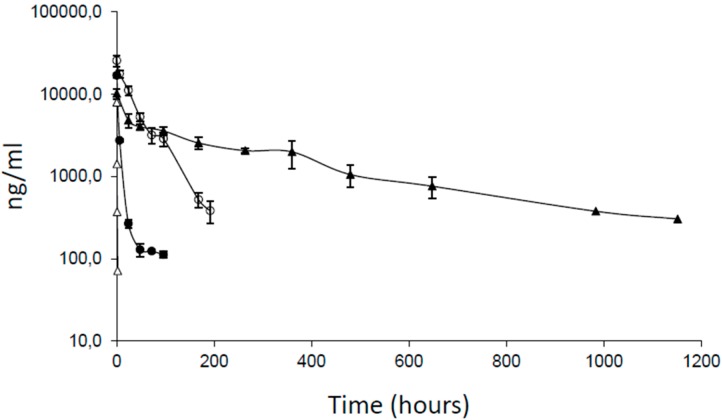
Plot of plasma concentrations of Trastuzumab and three related products obtained following single intravenous administration in Sprague-Dawley male rats (seven rats/group) of 2.0 mg/kg of Trastuzumab (▲), _Trast_Fab’ (∆), _Trast_F(ab’)_2_ (●) and _Trast_Fab’-Cys-PEG_(2 × 20 kDa)_ (○). Blood samples for determination of Trastuzumab or Fab fragment concentration were withdrawn from tail vein at pre-fixed times (from 0 to 1200 h) after product injection.

The pharmacokinetic features of the PEGylated _Trast_Fab’-Cys-PEG_(2 × 20 kDa)_ administered at 2.0 mg/kg by intravenous injections, was compared with those of _Trast_Fab’ and _Trast_F(ab’)_2_ administered under the same conditions and at the same doses. The whole Trastuzumab was also used in this study as a reference. As shown in Figure 4 and in Table 4, compared to the unPEGylated Fab fragments _Trast_Fab’ and _Trast_F(ab’)_2_, _Trast_Fab’-Cys-PEG_(2 × 20 kDa)_ showed a significantly increased half-life (α phase), which indeed went from 0.13 h (_Trast_Fab’) or 4.5 h (_Trast_F(ab’)_2_) to about 11 h for _Trast_Fab’-Cys-PEG_(2 × 20 kDa)_.

Concomitantly, the AUC (area under the curve) for _Trast_Fab’-Cys-PEG_(2 × 20 kDa)_ exhibited a 180-fold and 7.5-fold increase compared to _Trast_Fab’ and _Trast_F(ab’)_2_, respectively.

## 3. Discussion

Biotherapeutic proteins and monoclonal antibodies are an emerging class of new drugs with a number of pros and cons compared to traditional small molecules. Among the other advantages, proteins show in most cases an extremely high specificity and affinity for their targets; features that allow the use of smaller amounts compared to small molecules and that mostly improve the therapeutic index. On the other hand, large molecules are not captured by albumin like small organic compounds and more easily degraded by proteolytic enzymes; in addition, they are more prone to aggregation, can be immunogenic and poorly soluble at the therapeutic doses. Small proteins up to about 10 kDa also have reduced half-life in the blood stream because, independent from their degradation, they are rapidly excreted by glomerular filtration, a physiological process that ensure homeostasis of small sized proteins and biomolecules while hampering indiscriminate loss of larger ones. As a general principle, the larger is a biotherapeutic the more promptly it is retained in circulation, whereas those having low or intermediate molecular masses are only partly retained are filtered out to the urine. Monoclonal antibodies are large molecules (150 kDa) featuring half-lives of several days and do not require, in most cases, a stabilization process to increase the time they circulate in blood stream. On the other hand, despite sophisticated procedures for preventing immune response [25] and to increase shelf life are largely used to optimize the final drugs, they can still be partly immunogenic, have a general tendency to aggregate at high concentration, to precipitate and, given the very large surface, to stick to other proteins and tissues. PEGylation has been proposed as a general route to improve the pharmacokinetic and pharmacodynamics of antibodies [26] and of therapeutic proteins [27]. PEG mostly masks protein surface protecting them from the immune system and from proteases. At the same time it increases protein volume preventing or retarding release through kidneys. Chemical modifications, however, always alter to some extent protein properties and a compromise between increased molecular size and retained biological activity must be found to have a clinically useful drug. We have explored these aspects in this work by preparing several derivatives of the antibody Trastuzumab, one of the first mAbs to reach the market and one of the most important drug used to cure breast cancer. We have investigated the effects of PEG modification on the relative affinity of Trastuzumab, fragments for the target and the associated half-life *in vivo*. We thereby generated Trastuzumab Fabs, *M*w 50 kDa, F(ab’)_2_, *M*w 100 kDa, and a series of PEG derivatives having different *M*w and different sites of PEG attachment. The interest for antibody Fabs also derives from their potential to be used for the generation of Antibody Drug Conjugates (ADC), where the antibody portion mostly exert a tumour homing function, while the cell killing activity is driven by the conjugated cytotoxic moiety. In this case the ADCC activity of antibodies is negligible compared to that of the cytotoxic agent. We have found that Fab, Fab’ and F(ab’)_2_ retain a binding affinity similar to the whole antibody, whereas PEG attachment strongly influences recognition with HER2 receptor. In particular, the hardest affinity reduction (about 17-fold reduction considering the SPR data, (see Table 2)) was observed when 20 kDa PEG was attached to the N-terminus of the molecule, a site very close to the antigen binding site. This result was expected also in light of the very high antibody affinity, which is the result of highly sophisticated and complex interactions, so any minimal disturbance introduced by PEG, does affect recognition at kinetic or thermodymanic level. In this specific case, N-terminal PEGylation strongly affected both the *K*_a_ and the *K*_d_ (See Figures S7 and S11 and associated Tables) which then influenced the final *K*_D_.

A considerable loss of affinity was observed also when PEG at either 20 kDa or 40 kDa was attached on the C-terminus of the antibody Fab’ (about 8-fold or 5-fold reduction, respectively. See Table 2), suggesting that in this case, affinity reduction derived mostly from a less efficient diffusion rather than from a direct masking of the antibody binding site. Indeed, looking at the kinetic parameters of _Trast_Fab’-Cys-PEG_(2 × 20 kDa)_ in Figure S13 and the associated Table, Kon was the mostly affected parameter, passing from about 4.49 × 10^5^ to 7.04 × 10^4^ M**^−^**^1^·s**^−^**^1^ (High Density measure, see also Figure S7). This antibody derivative was however the one recovering at best the half-life, compared to the Fab’. This fragment indeed had a t_1/2_ β of only 0.36 h but increased about 100-fold upon PEGylation (See Table 4). The strong dependence on molecular size was also clearly seen by the much larger half-life of the F(ab’)_2_ (t_1/2_ β about 48 h), which has a *M*w of 100 kDa. Remarkably, the correlation between molecular size and half-life is almost linear between Fab’, F(ab’)_2_ and PEGylated Fab’, whereas the whole antibody, which is partly captured by neonatal Fc receptors (in humans) or more in general by Fc-γ receptors, is more rigid and possesses a more globular shape, shows an exponential increase in half-life. Also of notice, the short half-life of antibody fragments such as Fabs, coupled to the retained affinity is of particular importance for their use as molecular vectors in diagnostic [28,29] or to generate ADC, where the antibody only serves to address a potent cytotoxic toward tumor tissues. Indeed, the reduced size secures higher tissue penetration, higher dissociation rates from the molecular targets and short half-lives that dampen toxicity risks. To the best of our knowledge, this is the first study performed on so many different types of Herceptin derivatives reporting comparative analytical and binding data. Although still preliminary, the pharmacokinetic properties may also have relevance in providing a warning for the use of PEGylated antibody derivatives, whose pros and cons compared to the full size molecules have to be equally considered in a potential development program.

## 4. Materials and Methods

### 4.1. Media and Reagents

Monomethoxy-PEG-maleimide (10 and 20 kDa) and monomethoxy-PEG-propionaldehyde 20 kDa were from IRIS Biotech (Marktredwitz, Germany). Macrocap SP chromatographic resin, Sephacryl S200-HR resin, Superdex 75 resin and all reagents for Surface Plamon Resonance including sensor chips CM5, *N*-Hydroxysuccinimide (NHS) and 1-Ethyl-3-(3-dimethylaminopropyl) carbodiimide (EDC) were from GE Healthcare (Uppsala, Sweden).

Cysteamine, pepsin from porcine gastric mucosa, sodium cyanoborohydride, Goat Anti-Human IgG (Fab specific) conjugated to horseradish peroxidase, Bovine Serum Albumin, (3,3′,5,5′)-Tetramethylenbenzidine (TMB) substrate, Tween 20 and sulphuric acid were from Sigma-Aldrich (St. Louis, MO, USA). Recombinant Human ErbB2/HER2 Fc Chimera was purchased from R&D System (Minneapolis, MN, USA).

Sample solutions of Trastuzumab were obtained by pooling residual solutions (single samples all less than 50 μL) from discarded vials used for anti-cancer therapies. Vials were washed three times with the minimum amount of fresh deionized water and pooled. Antibody samples were characterized by 12% SDS-PAGE analysis under reducing and non-reducing conditions.

### 4.2. _Trast_Fab Preparation

The Fab fragment of Trastuzumab, hereafter _Trast_Fab (see Figure 1) was produced through proteolytic cleavage of the whole antibody using papain (Sigma-Aldrich). The reaction was performed in PBS buffer, pH 7.4, using a 1:100 (weight/weight, *w*/*w*) enzyme:substrate ratio. After pre-activation of papain with 5 mM l-cysteine for 10 min at room temperature, 2 mM EDTA and mAb were added and the final mixture was incubated for 90 min at 37 °C in a water bath. The reaction was stopped by addition of 25 mM Iodoacetamide, leaving the mixture for 20 min at room temperature (rt) in the dark to ensure complete alkylation of free cysteines.

The reaction mixture was purified through affinity chromatography onto a mAb Select Sure resin (GE Healthcare) to avoid co-elution of Fab with Fc [29]. For this purpose, the Fab fragment was eluted with PBS pH 7.4, while the Fc was removed with 100 mM Glycine, pH 2.7.

The collected Fab was concentrated using a 10,000 MWCO (molecular weight cut-off) Vivaspin (Sartorius, Goettingen, Germany) and subsequently purified by size-exclusion chromatography, applying the sample onto a Superdex75 10/300 GL column (GE Healthcare) and using PBS pH 7.4 as elution buffer. Next, the Fab fragment was finally concentrated in a 10,000 MWCO Vivaspin and quantified spectrophometrically at 280 nm using Nanodrop 2000c (ThermoFisher, Rockford, IL, USA). An ε_percent_ of 12 was adopted to calculate the concentration [30].

Purified fragments were analyzed by 12% SDS-PAGE under non-reducing and reducing conditions.

### 4.3. _Trast_F(ab’)_2_ Preparation

The F(ab’)_2_ of Trastuzumab (see Figure 1), hereafter _Trast_F(ab’)_2_*,* was prepared as follows. Trastuzumab at 4.0 mg/mL, was treated with pepsin from porcine gastric mucosa (Sigma-Aldrich) at 37 °C for 16 h, using a 20:1 antibody/pepsin *w*/*w* ratio in 0.1 M acetate buffer pH 4, 0.01 M EDTA [31,32]. _Trast_F(ab’)_2_ reaction mixture was then first dialyzed against 0.02 M acetate buffer pH 4.5 and then purified from the digestion mixture by cation exchange chromatography on a Macrocap SP chromatography column at a flow rate of 1.0 mL/min. A linear gradient of NaCl from 0 to 0.6 M in ten column volumes was applied to elute the material. Fractions containing _Trast_F(ab’)_2_ were concentrated by ultrafiltration and analyzed by SE-HPLC and 12% SDS-PAGE. SE chromatography analyses were performed using a Zorbax GF-250 (4.6 mm × 250 mm) column equilibrated at 0.3 mL/min in 0.063 M phosphate buffer pH 7.3, containing 3% (*v*/*v*) Isopropanol.

### 4.4. Preparation of the Fab’ of Trastuzumab

The Fab’ of Trastuzumab, hereafter _Trast_Fab’ (see Figure 1), was obtained by selective reduction of _Trast_F(ab’)_2_ with cysteamine [33]. _Trast_F(ab’)_2_, at 2.0 mg/mL in 0.1 M phosphate buffer, pH 6.0, 0.002 M EDTA, was incubated with cysteamine at a final concentration of 0.005 M. The reaction was carried out at room temperature for 16 h. Cysteamine was then removed by gel filtration chromatography using a Sephacryl S200-HR column equilibrated with 0.02 M acetate buffer pH 4.5, 0.01 M EDTA at a flow rate of 1.0 mL/min. Fractions containing _Trast_Fab’ were concentrated by ultrafiltration and analyzed by SE-HPLC and 12% SDS-PAGE. SE chromatography analyses were performed on a Zorbax GF-250 (4.6 mm × 250 mm) column in 0.063 M phosphate buffer pH 7.3, containing 3% (*v*/*v*) Isopropanol, at 25 °C, UV detection at 215 nm and applying a flow rate of 0.3 mL/min.

### 4.5. Preparation of Trastuzumab Fab’ Di-PEGylated on the Hinge Cysteine Residues

_Trast_Fab’ (see Figure 1) obtained by cysteamine reduction was purified by gel-filtration and reacted with MeO-PEG-Maleimide 10 or 20 kDa to obtain the derivatives di-PEGylated on the free cysteine residues present on the hinge region [34]. The reaction was typically carried out on 2.0 mg/mL _Trast_Fab’ solutions in 0.1 M phosphate buffer pH 6.0, 0.02 M EDTA, using a PEG/_Trast_Fab’ molar ratio of 7:1. The resulting solution was maintained under mild agitation for 6 h at room temperature. The di-PEGylated fragments, hereafter _Trast_Fab’-Cys-PEG_(2 × 20 kDa)_ and _Trast_Fab’-Cys-PEG_(2 × 10 kDa)_, were first dialyzed against 0.02 M acetate buffer pH 4.5 and then purified by cation exchange chromatography on a Macrocap SP chromatography column operating at a flow rate of 1.5 mL/min. PEGylated _Trast_Fab’s were eluted applying a linear gradient (10 column volumes) of NaCl from 0 to 0.2 M. Fractions containing the purified conjugated antibody fragments were analyzed by SE-HPLC and 12% SDS-PAGE. SE chromatography analyses were performed on a Zorbax GF-250 (4.6 mm × 250 mm) column in 0.063 M phosphate buffer pH 7.3, containing 3% (*v*/*v*) Isopropanol, at 25 °C, UV detection at 215 nm and applying a flow rate of 0.3 mL/min.

### 4.6. N-terminal Conjugation of _Trast_Fab

N-terminal PEG conjugation of _Trast_Fab (see Figure 1) was obtained by reductive alkylation using 20 kDa MeO-PEG-propionaldehyde.

To a 1.0 mg/mL _Trast_Fab solution in 0.1 M acetate buffer, pH 4.5, MeO-PEG-propionaldehyde was added in order to achieve a 1:2 Fab/PEG molar ratio. Under these conditions, PEG-aldehyde forms a Schiff base with the N-terminal amino group; the Shiff base is subsequently transformed into a stable secondary amine by mild reduction with sodium cyanoborohydride (NaBH_3_CN) [21]. NaBH_3_CN was added to the solution up to a final concentration of 0.04 M. The PEGylation reaction was carried out at room temperature for 16 h under gentle stirring. The conjugated Fab, hereafter _Trast_Fab-(N-Term)-PEG_20 kDa_, was first dialyzed against 0.02 M acetate buffer pH 4.5 and then purified by cation exchange chromatography on a Macrocap SP chromatography column operating at a flow rate of 1.5 mL/min. The conjugated Fab was eluted applying a 10 column volumes linear gradient of NaCl from 0 to 0.2 M. Fractions containing the purified _Trast_Fab-(N-Term)-PEG_20 kDa_ were analyzed by SE-HPLC and 12% SDS-PAGE. SE chromatography analyses were performed on a Zorbax GF-250 (4.6 mm × 250 mm) column in 0.063 M phosphate buffer pH 7.3, containing 3% (*v*/*v*) Isopropanol, at 25 °C, UV detection at 215 nm and applying a flow rate of 0.3 mL/min.

### 4.7. SDS-PAGE Analysis

SDS-PAGE was performed using a Bio-Rad Mini-Protean II vertical electrophoresis apparatus (Hercules, CA, USA), according to the method of Laemmli [35]. Antibody fragments and PEGylated compounds were mixed with Laemmli sample buffer (65.8 mM Tris-HCl, pH 6.8, 2.1% SDS, 26.3% glycerol, 0.01% bromophenol blue) and heated at 95 °C for 5 min, before being loaded onto the gel wells. A Bio-Safe Coomassie premixed solution was employed to stain the separated protein bands whereas iodine stain was used to detect PEGylated fragments.

### 4.8. Western Blotting Analysis of _Trast_Fab-(N-Term)-PEG_20 kDa_ and _Trast_Fab’-Cys-PEG_(2 × 10 kDa)_

Trastuzumab, _Trast_Fab-(N-Term)-PEG_20 kDa_ and _Trast_Fab’-Cys-PEG_(2 × 10 kDa)_ were reduced with 2-mercaptoethanol, separated by SDS-PAGE and then transferred to nitrocellulose membrane. After blocking the non-specific binding sites with 5% (*w*/*v*) bovine serum albumin, the membrane was incubated with anti-human IgG-Fab specific antibody conjugated to HRP (Sigma-Aldrich) which specifically recognize the Fab light chain. Immunoblots were visualized using the enhanced chemiluminescence detection kit (Thermoscientific, Rockford, IL, USA).

### 4.9. Analysis of Affinity by ELISA

Affinity of Fab fragments and PEGylated Fab fragments for the extracellular domain (ECD) of HER2/neu (also known as ErbB2/HER2) receptor was determined by an ELISA method performed in 96-well polystyrene microtiter plates. Plates were coated overnight at 4 °C with 100 µL/well of the recombinant ECD of human ErbB2/HER2 Fc Chimera at 0.5 μg/mL. The following day the plate was washed once with washing solution (PBS containing 0.1% *v*/*v* Tween 20). One hundred fifty microliters of the blocking buffer (PBS, 3% *w*/*v* BSA, 0.05% *v*/*v* Tween-20) was then added to each well and incubated with an orbital shaker at room temperature for 2 h. Wells were then gently washed with 200 µL of PBS-T buffer (PBS, 0.1% Tween-20) four times.

Fab fragments solutions in a range of concentrations from 6.25 to 3200 pM were added (100 µL) to each well, while PEGylated Fab fragments were tested in a range from 6.25 to 5000 pM. Each sample was prepared in PBS containing 0.5% (*w*/*v*) BSA and 0.05% (*w*/*v*) Tween-20.

The plate was then incubated for 1.5 h at room temperature. Sample solutions were then removed, and the wells washed four times with PBS-T buffer. One hundred microliters of goat anti-human IgG (Fab specific)-Peroxidase (1:40,000 dilution) was added into each well and incubated for 1 h at room temperature. The plate was then washed with PBS-T four times. One hundred microliters per well of TMB peroxidase substrate was added and after approximately 5 min the reaction was stopped by the addition of 100 μL 1N H_2_SO_4_ per well. Absorbance was measured at 450 nm on a microplate reader model Biorad 680 within 30 min.

### 4.10. Statistical Analysis

ELISA results are reported as mean of at least 4 values ± SD. Each single experiment was performed in parallel on the reference control (Trastuzumab). Statistical analysis of the difference between means was performed by Student’s *t* test.

Concentration–response curves were analyzed by using a non-linear curve fitting algorithm implemented in GraphPad (GraphPad Prism 4.0, San Diego, CA, USA), which provided *EC*_50_ values (concentration producing half-maximal response).

### 4.11. Analysis of Binding by SPR

All SPR binding assays were performed on a Biacore 3000 instrument (GE Healthcare). A CM5 sensor chip was functionalized with recombinant human ErbB2/Fc chimera receptor, running the Wizard method and applying a standard amine coupling chemistry [36,37]. Firstly, the sensor chip surface was activated with EDC/NHS. When ligand immobilization was complete, 1M ethanolamine, pH 8.5 was passed over the chip. Three channels at three different immobilization levels were prepared and named as Low (670 RU), Medium (1600) and High (3600) density, respectively.

The reference channel was functionalized with the Fc portion of Trastuzumab, purified from the previous Fab preparation. Signals from this channel were subtracted from the sample channels.

All binding assays were conducted at 25 °C, at a constant flow rate of 20 µL/min using HBS-EP (0.01 M HEPES pH 7.4, 0.15 M NaCl, 3 mM EDTA, 0.005% *v*/*v* Surfactant P20) as running buffer. All the samples were prepared in HBS-EP buffer and a total volume of 60 µL of each analyte solution was injected onto the surface at increasing concentrations providing a dose–response signal. The overall binding event was monitored for 550 s; the association step for 180 s while the dissociation step for 370 s and after each injection the chip surface was regenerated with NaOH using a range of concentrations comprised between 5 and 20 mM.

Fitting of binding data was performed by using BIAevaluation software version 4.1 (GE Healthcare, Uppsala, Sweden), adopting the 1:1 Langmuir binding model to extrapolate the kinetic parameters correlated to the association and dissociation rates of the ligand-analyte interaction. *k*_a_ stands for association constant, *k*_d_ stands for dissociation constant and *K*_D_ stands for apparent affinity constant.

The final dissociation constant value (*K*_D_) reported for each analyte was the average between the three *K*_D_ values extrapolated from every channel.

### 4.12. Pharmacokinetic Studies

Pharmacokinetic studies were performed in adult Sprague-Dawley male rats weighing about 250 grams obtained from Charles River (Calco, Lecco, Italy). All animal experiments were carried out in accordance with the provisions of the European Economic Community Council Directive 86/209 concerning the protection of animals used for experimental and other scientific purposes, recognized and adopted by the Italian Government with the approval decree D.M. No. 230/95-B.

Seven animals for each group received a single intravenous administration of 2.0 mg/kg of Trastuzumab or _Trast_Fab’-Cys-PEG_(2 × 20 kDa)_ or _Trast_F(ab’)_2_ dissolved at 5.0 mg/mL in 20 mM phosphate buffer, 0.14 M NaCl, pH 7.3. Blood samples (200 μL) of rats treated with trastuzumab were collected from tail vein at 10 min, 24, 48, 96, 168, 264, 360, 480, 648, 984 and 1152 h; the _Trast_Fab’-Cys-PEG_(2 × 20 kDa)_ group was sampled at 10 min, 24, 48, 72, 96, 168 and 192 h while the _Trast_F(ab’)_2_ group was sampled at 10 min, 24, 48, 72 and 96 h after products administration. Blood samples were collected into heparinized tubes; plasma was separated by centrifugation. Serum concentration of antibody and fragments was determined by ELISA, as described above.

## 5. Conclusions

In conclusion, we have here investigated the impact of different PEGylation strategies on the *in vivo* half-life and affinity for the target of Trastuzumab-derived Fabs. Data show that PEGylation largely improves half-life but, depending on the site and extent of PEGylation, affinity can be drastically reduced compared to the naked Fab or to the whole antibody. Data derived from this model antibody overall suggest that a fine balance between antibody size, affinity and choice of PEGylation site is needed to obtaining a sufficiently potent biomolecule with an acceptable half-life *in vivo*.

## Figures and Tables

**Table 1:** List of Trastuzumab derivatives used in this study. Table shows the predicted molecular weight (*M*w) of the molecules along with their corresponding *M*w as determined by SE-HPLC. * The Fab *M*w is seen as 36 kDa in our experimental conditions; ** The expected *M*w of product number 7 is predicted to be 90 kDa if PEG is attached on the N-terminus of both light and heavy chain; # Molecular volumes have been roughly calculated using the equation reported by Fee and Alstine [16] and using the *a* and *b* constants of albumin. Equation used: R_h,PEGprot_ = 0.82(M_r,prot_)^0.33^ + a + bM_r,PEGtot_. R_h,PEGprot_: hydrodynamic radius of the PEGylated protein in Angstrom; M_r,prot_: *M*w of the protein in Da; M_r,PEGtot_: *M*w of the PEG in Da.

Product Number	Product Description	Predicted *M*w (kDa)	Determined *M*w (SE-HPLC) (kDa)	Calculated Molecular Volume (nm^3^) ^#^
1	Trastuzumab	150	150	434
2	_Trast_Fab	50	36 *	163
3	_Trast_F(ab’)_2_	100	36 *	288
4	_Trast_Fab’	50	101	163
5	_Trast_Fab’-Cys-PEG_(2 × 10 kDa)_	70	490	700
6	_Trast_Fab’-Cys-PEG_(2 × 20 kDa)_	90	470	2000
7	_Trast_Fab-(N-Term)-PEG_20 kDa_	90 **	771	700

**Table 2:** Table of *EC*_50_ values of Trastuzumab and derivatives binding to HER2 receptor. *EC*_50_ values were determined by ELISA (GraphPad Prism ver.5.0).

Product	*EC*_50_ (nM)
Trastuzumab	0.228 ± 0.06
_Trast_Fab	0.459 ± 0.06
_Trast_Fab’	0.336 ± 0.08
_Trast_F(ab’)_2_	0.238 ± 0.03
_Trast_Fab-(N-Term)-PEG_20 kDa_	0.839 ± 0.02
_Trast_Fab’-Cys-PEG_(2 × 10 kDa)_	0.903 ± 0.13
_Trast_Fab’-Cys-PEG_(2 × 20 kDa)_	0.697 ± 0.14

**Table 3:** Average of kinetic parameters and apparent affinity constants of Trastuzumab and Fab derivatives obtained by surface plasmon resonance (SPR) technique using CM5 Chips with Immobilized ErbB2.

Product	K_a_ (×10^4^) M^−1^·s^−1^	K_d_ (×10^−5^) s^−1^	*K_D_* (k_d_/k_a_) nM
Trastuzumab	62.3	8.16	0.131
_Trast_Fab	59.9	24.2	0.405
_Trast_Fab’	57.9	17.2	0.297
_Trast_F(ab’)_2_	32.8	6.17	0.188
_Trast_Fab-(N-Term)-PEG_20 kDa_	7.71	37.2	4.82
_Trast_Fab’-Cys-PEG_(2 × 10 kDa)_	34.5	28.5	0.827
_Trast_Fab’-Cys-PEG_(2 × 20 kDa)_	7.03	15.8	2.24

**Table 4:** Table of the main pharmacokinetic parameters calculated after *i.v.* administration of Trastuzumab, _Trast_F(ab’)_2_, _Trast_Fab’ and _Trast_Fab’-Cys-PEG_(2 × 20 kDa)_.

Product	Dose	C max (ng/mL)	AUC (ng/mL h)	t_1/2_ α (h)	t_1/2_ β (h)
Trastuzumab	2.0 mg/kg	26,315.8 *	1,694,759.1 (1152 h)	22.1	288.8
_Trast_F(ab’)_2_	2.0 mg/kg	18,947.4 *	116,293.6 (96 h)	4.5	48.1
_Trast_Fab’	2.0 mg/kg	25,0002 ^+^	4985.3 (2 h)	0.13	0.36
_Trast_Fab’-Cys-PEG_(2 × 20 kDa)_	2.0 mg/kg	26,315.8 *	896,387.8 (192 h)	11	35.4

* Estimated value as the administered dose compared to total blood volume (19.0 mL), 8% of the rat weight; _+_ Estimated value as the administered dose compared to total blood volume (22.4 mL), 8% of the rat weight.

## Supplementary Materials

Supplementary materials can be found at http://www.mdpi.com/1422-0067/17/4/491/s1.
