# Supplementary material for: Pegylated Trastuzumab Fragments Acquire an Increased in Vivo Stability but Show a Largely Reduced Affinity for the Target Antigen
Source: Int J Mol Sci. 2016 Apr 1;17(4):491. doi: 10.3390/ijms17040491 (PMC4848947; doi:10.3390/ijms17040491)
Supplement: Supplementary file 1 [file ijms-17-00491-s001.pdf]

Supplementary Material: Pegylated Trastuzumab Fragments Acquire an Increased *in Vivo* Stability but Show a Largely Reduced Affinity for the Target Antigen

Fabio Selis, Giuseppina Focà, Annamaria Sandomenico, Carla Marra, Concetta Di Mauro, Gloria Saccani Jotti, Silvia Scaramuzza, Annalisa Politano, Riccardo Sanna, Menotti Ruvo and Giancarlo Tonon

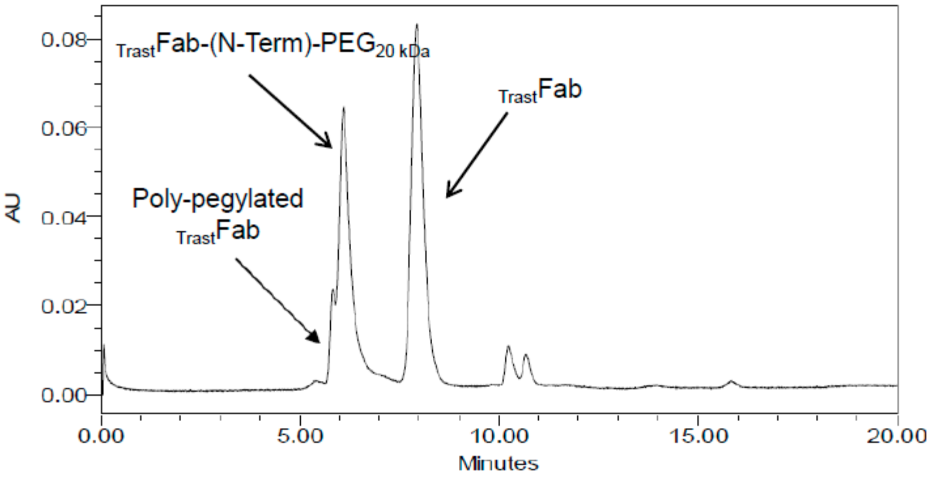

Figure S1. HPLC analysis of the PEGylation mixture of $_{Trast}$Fab (1.0 mg/mL) with mPEG-aldehyde 20 kDa in 0.1 M acetate buffer pH 4.5 after 16 h reaction at room temperature. The molar ratio PEG/$_{Trast}$Fab was 2:1. Reaction mixture was analysed by SE-HPLC using a Zorbax GF-250 column (4.6 mm × 250 mm). SE-HPLC analysis were performed in 0.063 M phosphate buffer pH 7.3, 3% (*w*/*w*) Isopropanol, at 45 °C, UV detection at 215 nm and with a flow rate of 0.3 mL/min.

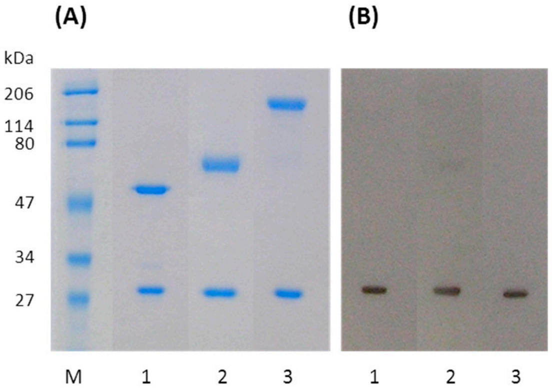

Figure S2. SDS-PAGE (10% separating gel) (**A**) and Western Blotting (**B**) analysis of: Trastuzumab (lane 1); $_{Trast}$Fab-(N-Term)-PEG$_{20\ kDa}$ (lane 2), $_{Trast}$Fab′-Cys-PEG$_{(2\times20\ kDa)}$ (lane 3) and protein standards (lane M). All samples were analyzed after extensive reduction by 2-mercaptoethanol before loading.

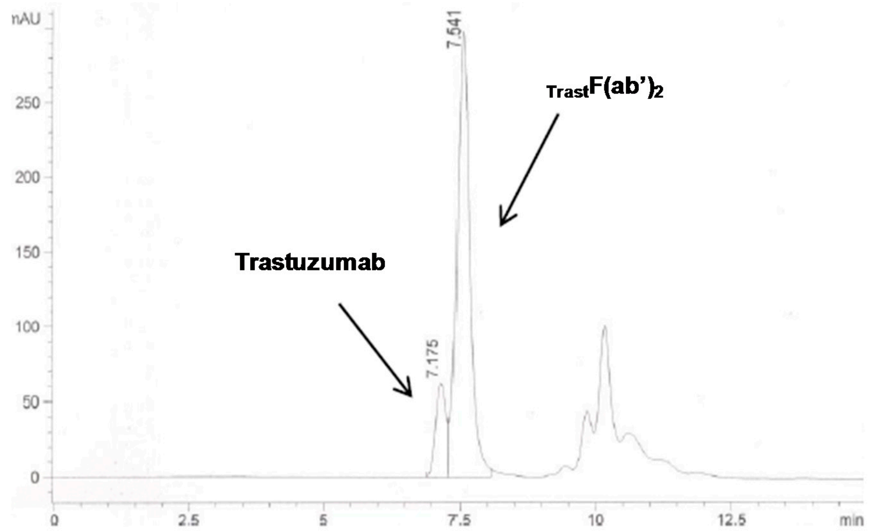

Figure S3. SE-HPLC analysis of Trastuzumab digestion with pepsin from porcine gastric mucosa after 16 hours reaction. The digestion reaction was carried out at 37 °C, 4.0 mg/mL. Trastuzumab concentration and a 20:1 antibody/pepsin weight ratio was used in 0.1 M acetate buffer pH 4, 0.01 M EDTA. HPLC analysis was performed on a Zorbax GF-250 column (4.6 mm × 250 mm) in 0.063 M phosphate buffer pH 7.3, 3% (*w*/*w*) Isopropanol, at 45 °C, UV detection at 215 nm and with a flow rate of 0.3 mL/min.

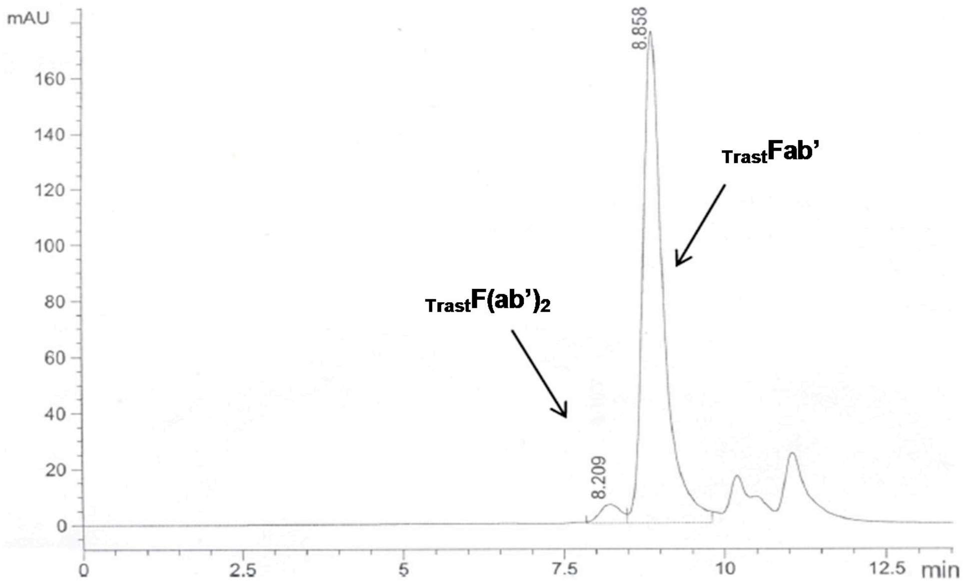

Figure S4. SE-HPLC analysis of ${}_{Trast}F(ab')_2$ reduction with Cysteamine after 16 h reaction. The reduction was carried out at 37 °C, 2.0 mg/mL ${}_{Trast}F(ab')_2$ concentration and 0.05 M Cysteamine in 0.1 M phosphate buffer pH 6.0, 0.02 M EDTA. HPLC analysis was performed on a Zorbax GF-250 column (4.6 mm × 250 mm) in 0.063 M phosphate buffer pH 7.3, 3% (*w*/*w*) Isopropanol, at 45 °C, UV detection at 215 nm and with a flow rate of 0.3 mL/min.

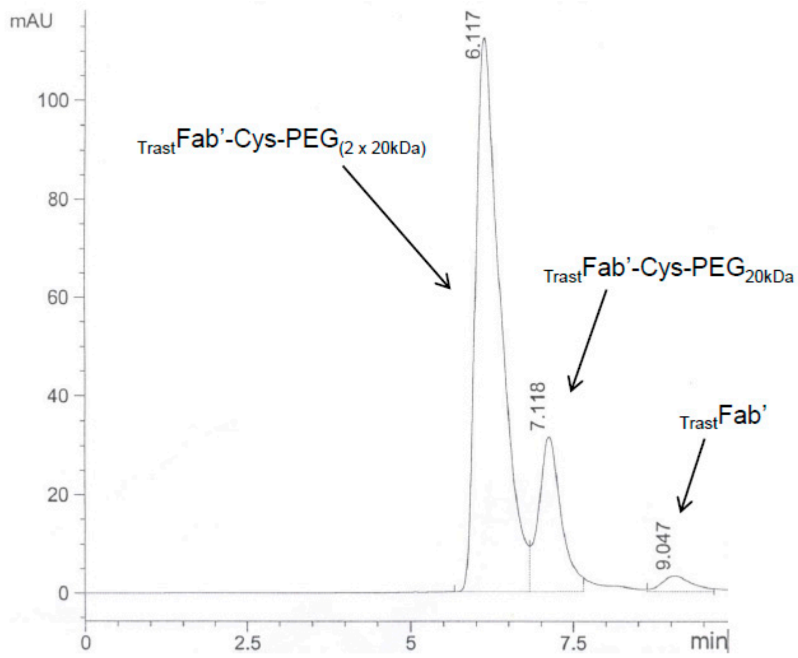

Figure S5. HPLC analysis of PEGylation mixture of $_{Trast}$Fab′ (2.0 mg/mL) with mPEG-maleimide 20 kDa in 0.1 M phosphate buffer pH 6.0, 0.02 M EDTA after 6 h reaction at room temperature. Reaction mixture was analysed by SE-HPLC using a Zorbax GF-250 column (4.6 mm × 250 mm). SE-HPLC analysis was performed in 0.063 M phosphate buffer pH 7.3, 3% (*w*/*w*) Isopropanol, at 45 °C; UV detection at 215 nm with a flow rate of 0.3 mL/min.

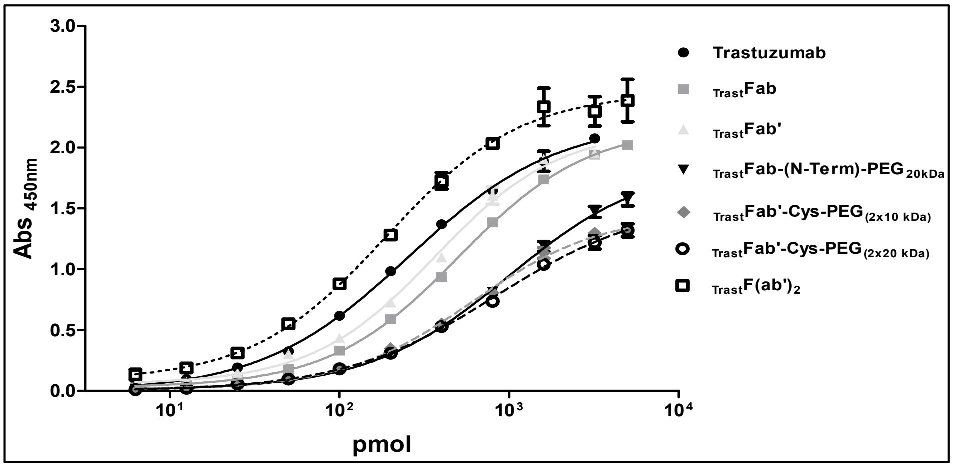

Figure S6. Superimposed ELISA binding curves of Trastuzumab and derivatives to recombinant human ErbB2. Wells were coated with 0.5 μg/mL of receptor and analytes were used at increasing concentrations, ranging from 6.25 up to 5000 pM, except for Trastuzumab and $_{Trast}$Fab′, used up to 3200 pM. Curves were fitted using GraphPad Prism software (ver. 5.0).

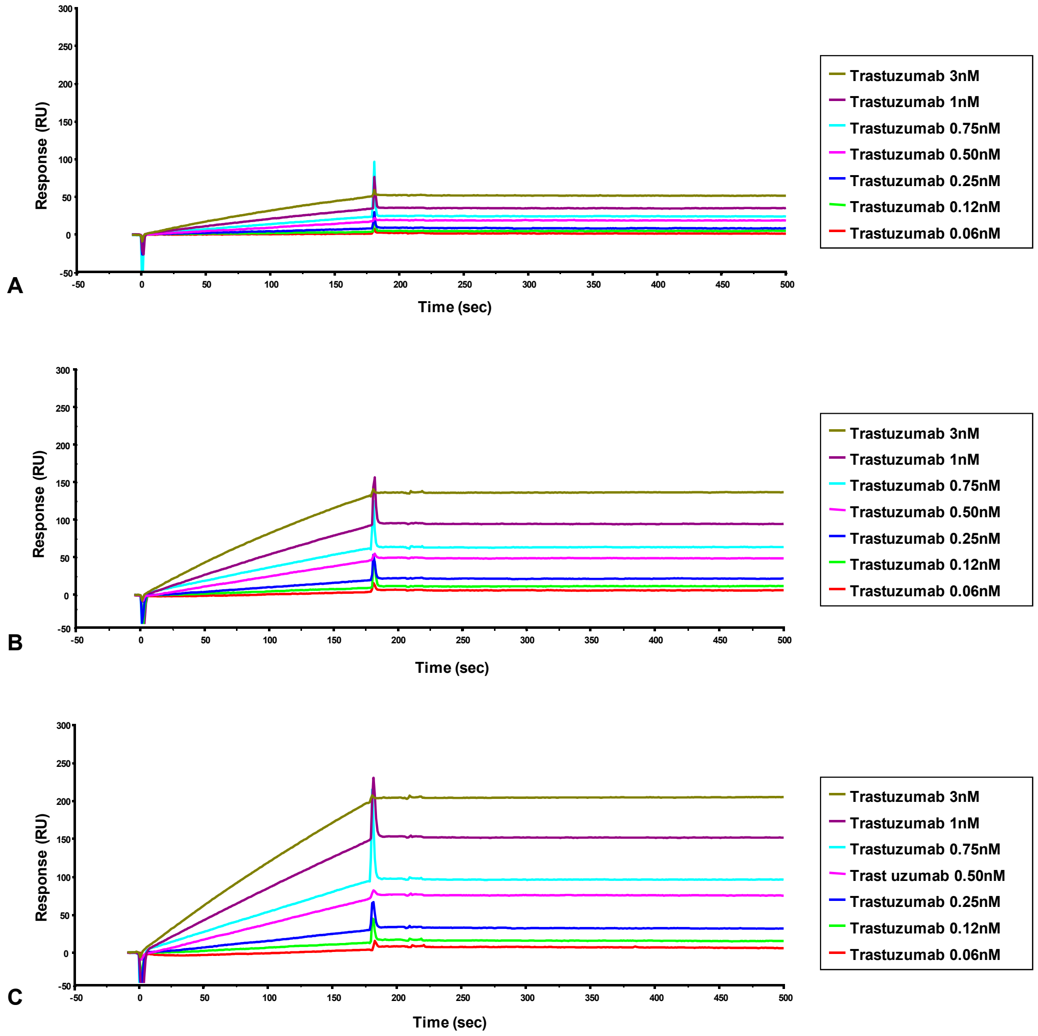

Figure S7. *Cont.*

D	Low Density			Medium Density			High Density		
Trastuzumab	K_a (1/Ms)	K_d (1/s)	K_D (M)	K_a (1/Ms)	K_d (1/s)	K_D (M)	K_a (1/Ms)	K_d (1/s)	K_D (M)
0.06 nM	2.97×10^{5}	5.37×10^{-4}	1.81×10^{-9}	1.11×10^{6}	2.37×10^{-4}	2.14×10^{-10}	2.50×10^{5}	7.46×10^{-4}	2.99×10^{-9}
0.12 nM	1.86×10^{5}	4.88×10^{-4}	2.62×10^{-9}	3.32×10^{5}	5.25×10^{-5}	1.58×10^{-10}	2.15×10^{5}	2.22×10^{-4}	1.03×10^{-9}
0.25 nM	4.37×10^{5}	6.06×10^{-4}	1.39×10^{-9}	3.29×10^{5}	9.94×10^{-5}	3.02×10^{-10}	7.37×10^{4}	1.93×10^{-4}	2.62×10^{-9}
0.50 nM	4.15×10^{4}	4.92×10^{-6}	1.19×10^{-10}	1.08×10^{5}	1.17×10^{-5}	1.09×10^{-10}	3.27×10^{4}	7.57×10^{-6}	2.32×10^{-10}
0.75 nM	1.09×10^{5}	6.99×10^{-5}	6.44×10^{-10}	1.23×10^{6}	2.10×10^{-5}	1.71×10^{-11}	5.28×10^{5}	1.08×10^{-5}	2.05×10^{-11}
1.00 nM	1.55×10^{6}	6.94×10^{-5}	4.48×10^{-11}	1.16×10^{6}	1.17×10^{-5}	1.01×10^{-11}	8.70×10^{5}	1.62×10^{-5}	1.86×10^{-11}
3.00 nM	1.10×10^{6}	5.95×10^{-5}	5.42×10^{-11}	1.04×10^{6}	2.03×10^{-5}	1.96×10^{-11}	7.40×10^{5}	2.22×10^{-5}	3.00×10^{-11}

Figure S7. (**A**–**D**) Sensorgrams overlay of Trastuzumab binding to recombinant ErbB2 protein immobilized onto a CM5 chip at a low (**A**), medium (**B**) and high (**C**) density. Binding assays were performed at 25 °C and at a constant flow rate of 20 μL/min, using HBS-EP as running buffer. Table of kinetic and thermodynamic parameters (**D**).

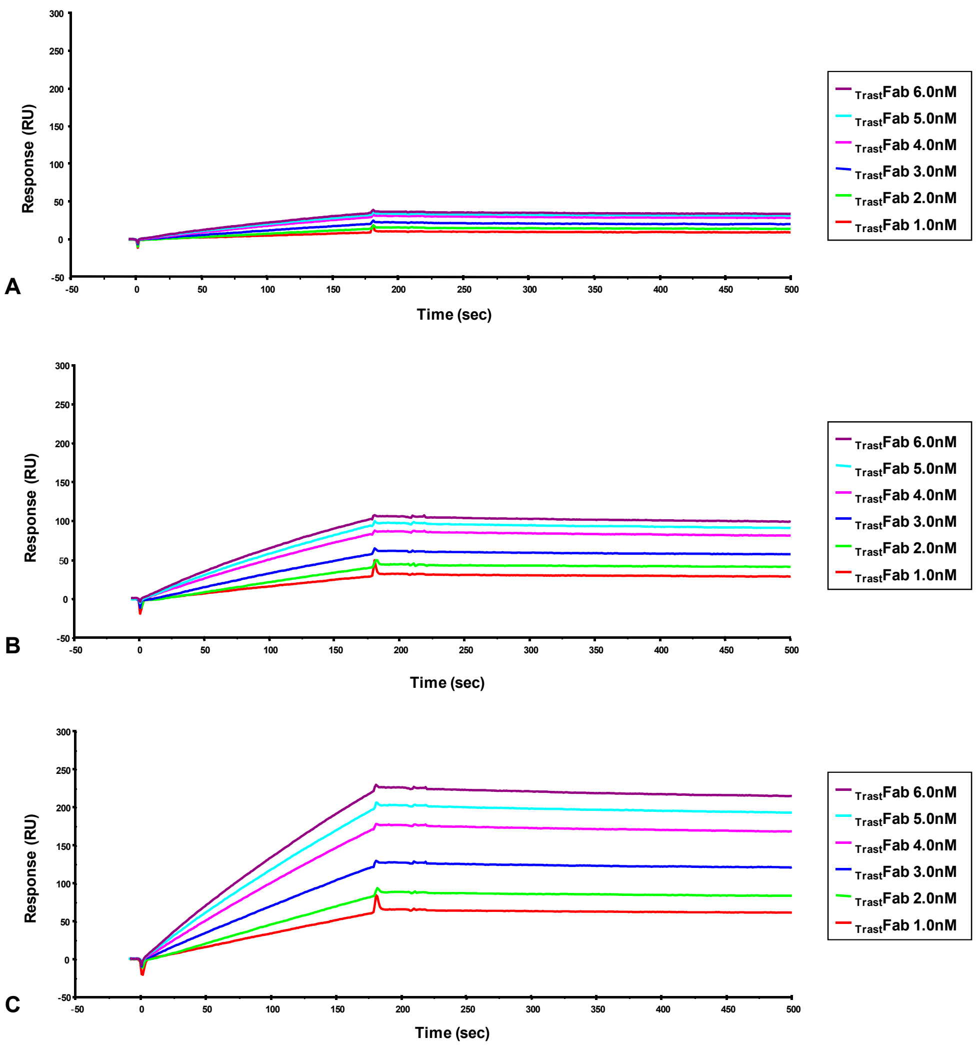

Figure S8. *Cont.*

D	Low Density			Medium Density			High Density		
$_{Trast}$Fab	K_a (1/Ms)	K_d (1/s)	K_D (M)	K_a (1/Ms)	K_d (1/s)	K_D (M)	K_a (1/Ms)	K_d (1/s)	K_D (M)
1 nM	1.05×10^{5}	5.62×10^{-4}	5.33×10^{-9}	1.69×10^{6}	3.05×10^{-4}	1.81×10^{-10}	1.87×10^{5}	1.90×10^{-4}	1.02×10^{-9}
2 nM	1.50×10^{5}	3.39×10^{-4}	2.26×10^{-9}	5.02×10^{5}	1.82×10^{-4}	3.62×10^{-10}	4.10×10^{5}	1.61×10^{-4}	3.92×10^{-10}
3 nM	1.42×10^{6}	3.85×10^{-4}	2.70×10^{-10}	6.11×10^{5}	2.45×10^{-4}	4.01×10^{-10}	4.48×10^{5}	1.84×10^{-4}	4.11×10^{-10}
4 nM	9.56×10^{5}	3.91×10^{-4}	4.09×10^{-10}	5.64×10^{5}	2.19×10^{-4}	3.8×10^{-10}	3.46×10^{5}	1.71×10^{-4}	4.94×10^{-10}
5 nM	6.16×10^{5}	2.83×10^{-4}	4.60×10^{-10}	6.60×10^{5}	2.26×10^{-4}	3.42×10^{-10}	3.78×10^{5}	1.64×10^{-4}	4.34×10^{-10}
6 nM	8.50×10^{5}	2.74×10^{-4}	3.23×10^{-10}	6.57×10^{5}	2.21×10^{-4}	3.36×10^{-10}	4.11×10^{5}	1.89×10^{-4}	4.60×10^{-10}

Figure S8. (**A**–**D**) Sensorgrams overlay of $_{Trast}$Fab fragment binding to recombinant ErbB2 protein immobilized onto a CM5 chip at a low (**A**), medium (**B**) and high (**C**) density. Fab was generated through papain cleavage of the full-size antibody. Binding assays were performed at 25 °C and at a constant flow rate of 20 μL/min, using HBS-EP as running buffer. Table of kinetic and thermodynamic parameters (**D**).

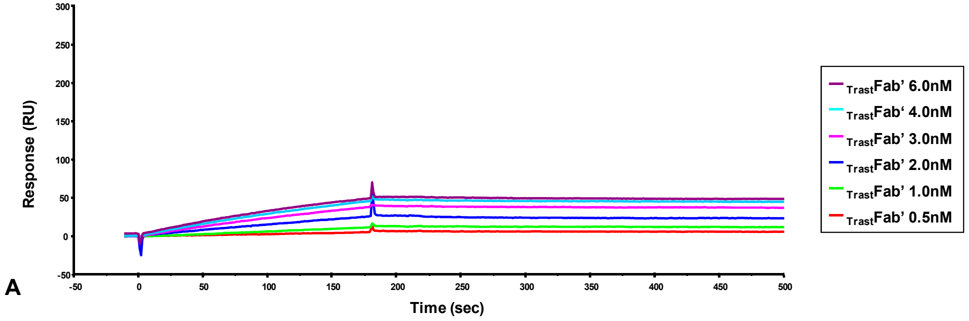

A

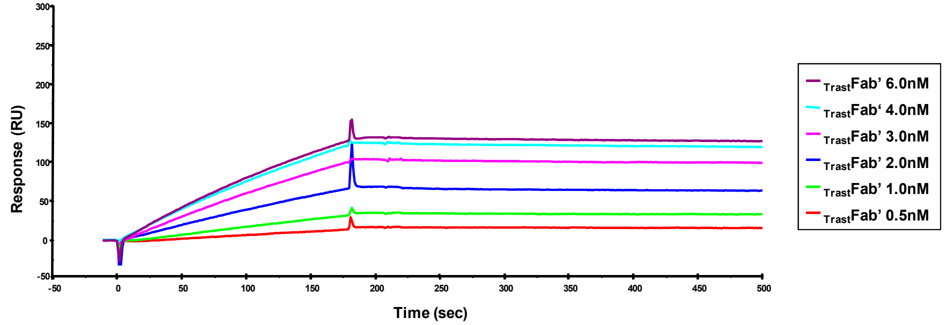

B

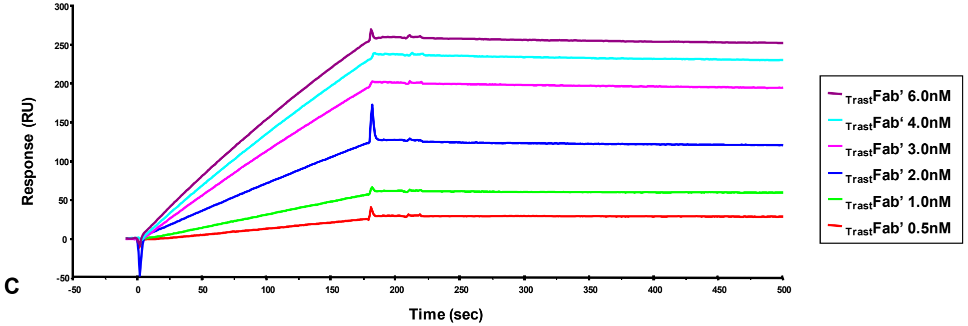

C

Figure S9. *Cont.*

D	Low Density	Medium Density	High Density

TrastFab′	K_a (1/Ms)	K_d (1/s)	K_D (M)	K_a (1/Ms)	K_d (1/s)	K_D (M)	K_a (1/Ms)	K_d (1/s)	K_D (M)
0.5 nM	3.92×10^5	3.21×10^{-4}	8.19×10^{-10}	9.40×10^4	1.26×10^{-4}	1.34×10^{-9}	1.97×10^5	2.81×10^{-5}	1.43×10^{-10}
1 nM	5.64×10^4	4.74×10^{-5}	8.40×10^{-10}	1.60×10^5	2.36×10^{-4}	1.47×10^{-9}	3.35×10^5	1.69×10^{-4}	5.05×10^{-10}
2 nM	6.39×10^5	4.52×10^{-4}	7.08×10^{-10}	8.70×10^5	2.02×10^{-4}	2.32×10^{-10}	4.33×10^5	1.65×10^{-4}	3.81×10^{-10}
3 nM	1.10×10^6	1.77×10^{-4}	1.61×10^{-10}	8.57×10^5	1.54×10^{-4}	1.80×10^{-10}	4.10×10^5	1.15×10^{-4}	2.80×10^{-10}
4 nM	9.53×10^5	1.77×10^{-4}	1.86×10^{-10}	7.42×10^5	1.25×10^{-4}	1.68×10^{-10}	4.47×10^5	1.19×10^{-4}	2.66×10^{-10}
6 nM	6.54×10^5	2.12×10^{-4}	3.24×10^{-10}	6.13×10^5	1.25×10^{-4}	2.04×10^{-10}	4.15×10^5	1.00×10^{-4}	2.41×10^{-10}

Figure S9. (**A**–**D**) Sensorgrams overlay of TrastFab′ fragment binding to recombinant ErbB2 protein immobilized onto a CM5 chip at a low (**A**), medium (**B**) and high (**C**) density. Fab′ was generated through specific reduction of $F(ab')_2$. Binding assays were performed at 25 °C and at a constant flow rate of 20 μL/min, using HBS-EP as running buffer. Table of kinetic and thermodynamic parameters (**D**).

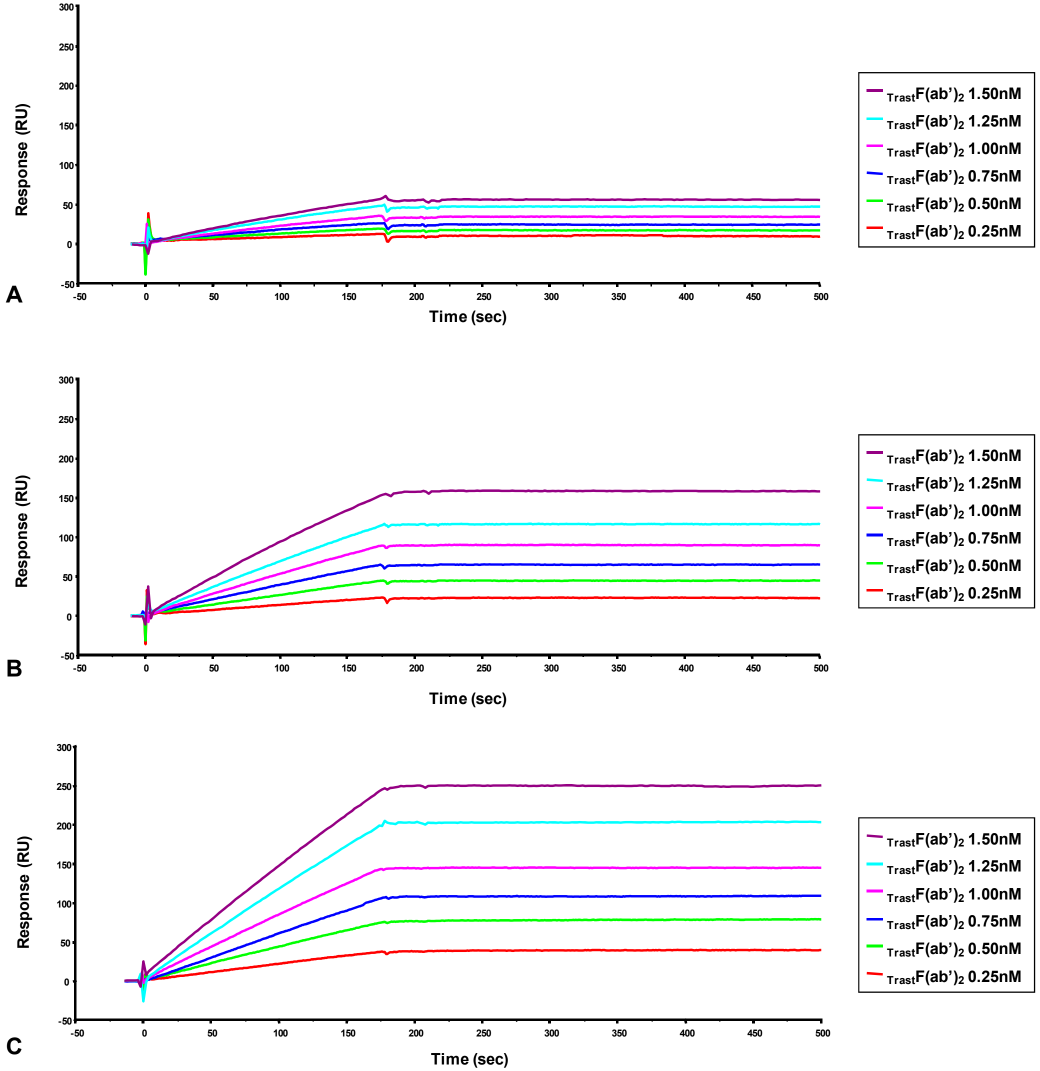

Figure S10. *Cont.*

**D**	**Low Density**			**Medium Density**			**High Density**		
${}_{Trast}F(ab')_2$	K_a (1/Ms)	K_d (1/s)	K_D (M)	K_a (1/Ms)	K_d (1/s)	K_D (M)	K_a (1/Ms)	K_d (1/s)	K_D (M)
0.25 nM	1.25×10^{5}	1.90×10^{-4}	1.51×10^{-9}	5.42×10^{4}	1.38×10^{-5}	2.54×10^{-10}	5.70×10^{4}	2.32×10^{-5}	4.07×10^{-10}
0.50 nM	1.27×10^{5}	1.78×10^{-4}	1.40×10^{-9}	2.58×10^{4}	9.43×10^{-6}	3.65×10^{-10}	1.83×10^{5}	1.30×10^{-4}	7.09×10^{-1}
0.75 nM	2.00×10^{5}	2.57×10^{-4}	1.28×10^{-9}	1.08×10^{5}	1.15×10^{-5}	1.06×10^{-10}	7.89×10^{4}	2.39×10^{-6}	3.03×10^{-11}
1.00 nM	1.56×10^{5}	1.57×10^{-5}	1.00×10^{-10}	9.75×10^{5}	1.27×10^{-5}	1.30×10^{-11}	3.90×10^{5}	1.12×10^{-5}	2.88×10^{-11}
1.25 nM	1.14×10^{6}	1.56×10^{-4}	1.37×10^{-10}	9.57×10^{4}	2.45×10^{-6}	2.56×10^{-11}	6.17×10^{5}	2.83×10^{-5}	4.59×10^{-11}
1.50 nM	8.97×10^{4}	4.37×10^{-5}	4.87×10^{-10}	8.91×10^{5}	1.53×10^{-5}	1.72×10^{-11}	5.99×10^{5}	1.06×10^{-5}	1.77×10^{-11}

Figure S10. (**A**–**D**) Sensorgrams overlay of ${}_{Trast}F(ab')_2$ fragment binding to recombinant ErbB2 protein immobilized onto a CM5 chip at a low (**A**), medium (**B**) and high (**C**) density. $F(ab')_2$ was generated through pepsin cleavage of the full-size antibody. Binding assays were performed at 25 °C and at a constant flow rate of 20 μL/min, using HBS-EP as running buffer. Table of kinetic and thermodynamic parameters (**D**).

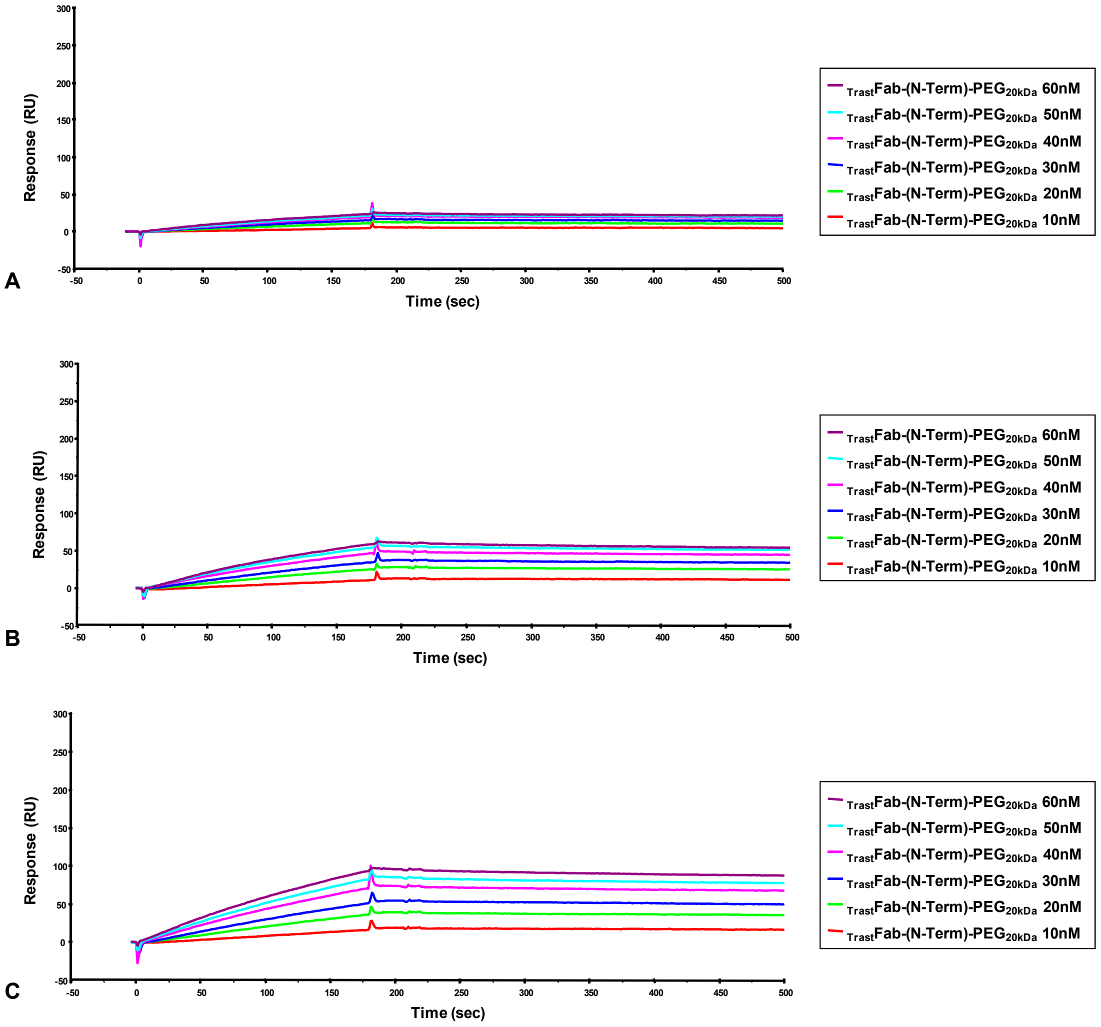

Figure S11. *Cont.*

D	Low Density			Medium Density			High Density		
$_{Trast}$Fab-(N-Term)-PEG$_{20\ kDa}$	K_a (1/Ms)	K_d (1/s)	K_D (M)	K_a (1/Ms)	K_d (1/s)	K_D (M)	K_a (1/Ms)	K_d (1/s)	K_D (M)
10 nM	1.94×10^{4}	2.80×10^{-4}	1.44×10^{-8}	2.51×10^{4}	1.96×10^{-4}	7.82×10^{-9}	5.38×10^{4}	3.09×10^{-4}	5.74×10^{-9}
20 nM	2.23×10^{4}	7.71×10^{-4}	3.46×10^{-8}	7.54×10^{4}	3.38×10^{-4}	4.48×10^{-9}	5.89×10^{4}	3.31×10^{-4}	5.62×10^{-9}
30 nM	8.46×10^{4}	3.45×10^{-4}	4.08×10^{-8}	6.64×10^{4}	3.90×10^{-4}	5.88×10^{-9}	6.82×10^{4}	2.21×10^{-4}	3.24×10^{-9}
40 nM	1.09×10^{5}	3.41×10^{-4}	3.12×10^{-9}	1.06×10^{5}	2.69×10^{-4}	2.53×10^{-9}	8.28×10^{4}	2.63×10^{-4}	3.18×10^{-9}
50 nM	8.43×10^{4}	4.11×10^{-4}	4.88×10^{-9}	8.48×10^{4}	3.46×10^{-4}	4.08×10^{-9}	6.25×10^{4}	3.31×10^{-4}	5.29×10^{-9}
60 nM	9.17×10^{4}	4.70×10^{-4}	5.12×10^{-9}	9.41×10^{4}	4.21×10^{-4}	4.47×10^{-9}	6.49×10^{4}	3.27×10^{-4}	5.04×10^{-9}

Figure S11. (**A**–**D**) Sensorgrams overlay of $_{Trast}$Fab-(N-Term)-PEG$_{20\ kDa}$ derivative binding to recombinant ErbB2 protein immobilized onto a CM5 chip at a low (**A**), medium (**B**) and high (**C**) density. The Fab fragment was coupled with a single 20 kDa PEG tail at the N-terminus. Binding assays were performed at 25 °C and at a constant flow rate of 20 μL/min, using HBS-EP as running buffer. Table of kinetic and thermodynamic parameters (**D**).

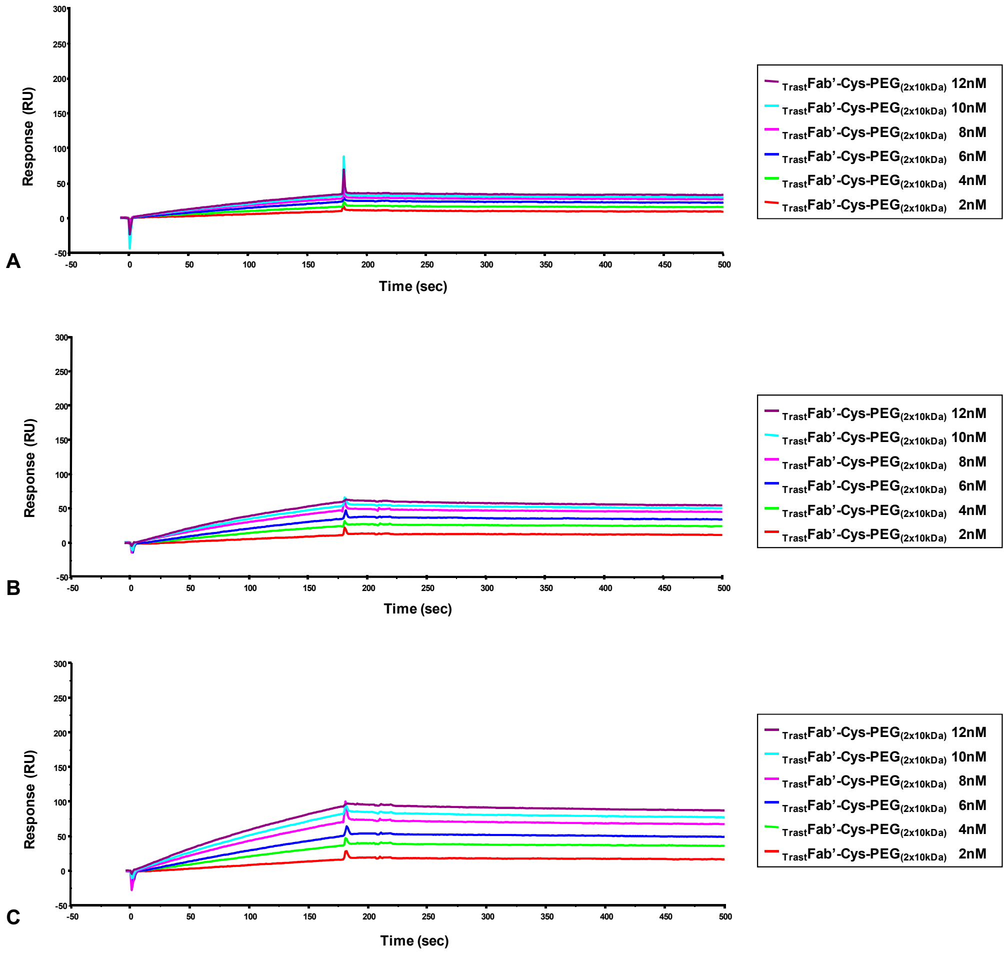

Figure S12. *Cont.*

D	Low Density			Medium Density			High Density		
$_{Trast}$Fab′-Cys-PEG$_{(2 \times 10\ kDa)}$	K_a (1/Ms)	K_d (1/s)	K_D (M)	K_a (1/Ms)	K_d (1/s)	K_D (M)	K_a (1/Ms)	K_d (1/s)	K_D (M)
2 nM	6.23×10^{4}	6.34×10^{-4}	1.02×10^{-8}	7.66×10^{4}	4.01×10^{-4}	5.24×10^{-9}	3.77×10^{4}	3.23×10^{-4}	8.56×10^{-9}
4 nM	6.62×10^{4}	3.22×10^{-4}	4.86×10^{-9}	5.09×10^{5}	2.94×10^{-4}	5.77×10^{-10}	2.67×10^{5}	2.40×10^{-4}	8.99×10^{-10}
6 nM	4.70×10^{5}	3.22×10^{-4}	6.85×10^{-10}	4.16×10^{5}	2.32×10^{-4}	5.58×10^{-10}	4.05×10^{5}	2.45×10^{-4}	6.05×10^{-10}
8 nM	5.48×10^{5}	2.54×10^{-4}	4.64×10^{-10}	4.12×10^{5}	2.10×10^{-4}	5.10×10^{-10}	3.25×10^{5}	1.57×10^{-4}	4.83×10^{-10}
10 nM	5.71×10^{5}	3.26×10^{-4}	5.71×10^{-10}	3.89×10^{5}	2.05×10^{-4}	5.27×10^{-10}	3.12×10^{5}	1.83×10^{-4}	5.86×10^{-10}
12 nM	6.67×10^{5}	3.27×10^{-4}	4.90×10^{-10}	3.74×10^{5}	2.43×10^{-4}	6.50×10^{-10}	3.00×10^{5}	2.17×10^{-4}	7.24×10^{-10}

Figure S12. (**A**–**D**) Sensorgrams overlay of $_{Trast}$Fab′-Cys-PEG$_{(2 \times 10\ kDa)}$ derivative binding to recombinant ErbB2 protein immobilized onto a CM5 chip at a low (**A**), medium (**B**) and high (**C**) density. The Fab′ fragment was coupled with two 10 kDa PEG tails at the free cysteines on the heavy chain C-terminus. Binding assays were performed at 25 °C and at a constant flow rate of 20 μL/min, using HBS-EP as running buffer. Table of kinetic and thermodynamic parameters (**D**).

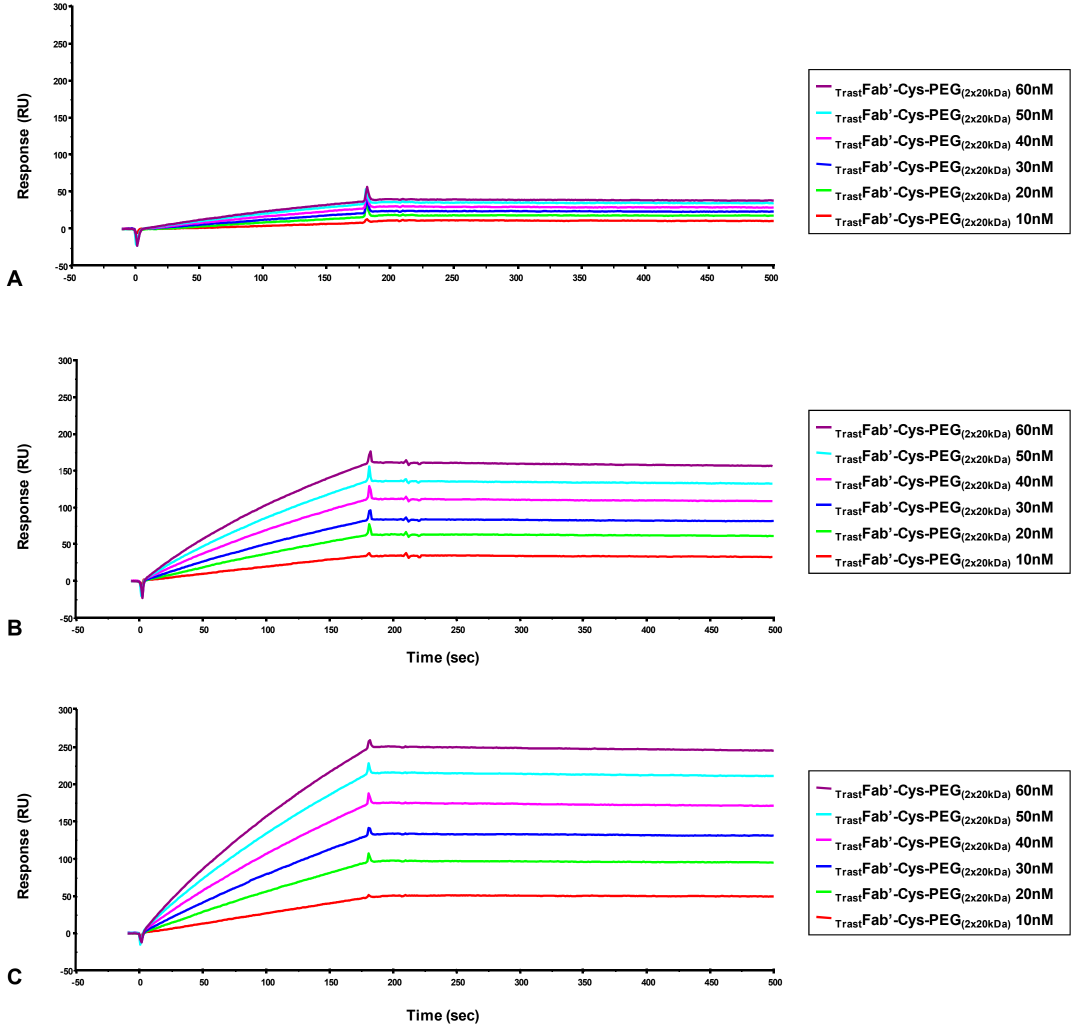

Figure S12. *Cont.*

D	Low Density			Medium Density			High Density		
$_{Trast}$Fab′-Cys-PEG$_{(2 \times 20\ kDa)}$	K_a (1/Ms)	K_d (1/s)	K_D (M)	K_a (1/Ms)	K_d (1/s)	K_D (M)	K_a (1/Ms)	K_d (1/s)	K_D (M)
10 nM	3.11×10^4	7.37×10^{-4}	2.37×10^{-8}	1.14×10^5	3.10×10^{-4}	2.72×10^{-9}	4.60×10^4	1.84×10^{-4}	4.00×10^{-9}
20 nM	3.86×10^4	4.73×10^{-5}	1.22×10^{-9}	8.15×10^4	1.11×10^{-4}	1.36×10^{-9}	9.75×10^4	7.40×10^{-5}	7.59×10^{-10}
30 nM	6.77×10^4	7.10×10^{-5}	1.05×10^{-9}	9.63×10^4	$1.1 \times 10^{-4}6$	1.20×10^{-9}	7.34×10^4	4.26×10^{-5}	5.81×10^{-10}
40 nM	5.49×10^4	2.22×10^{-4}	4.04×10^{-9}	8.19×10^4	1.07×10^{-4}	1.31×10^{-9}	7.29×10^4	8.60×10^{-5}	1.18×10^{-9}
50 nM	5.33×10^4	1.72×10^{-4}	3.23×10^{-9}	8.55×10^4	1.60×10^{-4}	1.87×10^{-9}	6.73×10^4	7.85×10^{-5}	1.17×10^{-9}
60 nM	5.72×10^4	1.27×10^{-4}	2.22×10^{-9}	8.00×10^4	1.19×10^{-4}	1.49×10^{-9}	6.54×10^4	7.39×10^{-5}	1.13×10^{-9}

Figure S13. (**A**–**D**) Sensorgrams overlay of $_{Trast}$Fab′-Cys-PEG$_{(2 \times 20\ kDa)}$ derivative binding to recombinant ErbB2 protein immobilized onto a CM5 chip at a low (**A**), medium (**B**) and high (**C**) density. The Fab′ fragment was coupled with two 20 kDa PEG tails at the free cysteines on the heavy chain C-terminus. Binding assays were performed at 25 °C and at a constant flow rate of 20 μL/min, using HBS-EP as running buffer. Table of kinetic and thermodynamic parameters (**D**).
